# Molecular mechanisms in uterine epithelium during trophoblast binding: the role of small GTPase RhoA in human uterine Ishikawa cells

**DOI:** 10.1186/1743-1050-2-4

**Published:** 2005-03-09

**Authors:** Carola Heneweer, Martina Schmidt, Hans-Werner Denker, Michael Thie

**Affiliations:** 1 Institute of Anatomy, University Hospital Essen, Germany; 2 Institute of Pharmacology, University Hospital Essen, Germany; 3 Stiftung caesar, Bonn, Germany

## Abstract

**Background:**

Embryo implantation requires that uterine epithelium develops competence to bind trophoblast to its apical (free) poles. This essential element of uterine receptivity seems to depend on a destabilisation of the apico-basal polarity of endometrial epithelium. Accordingly, a reorganisation of the actin cytoskeleton regulated by the small GTPase RhoA plays an important role in human uterine epithelial RL95-2 cells for binding of human trophoblastoid JAR cells. We now obtained new insight into trophoblast binding using human uterine epithelial Ishikawa cells.

**Methods:**

Polarity of Ishikawa cells was investigated by electron microscopy, apical adhesiveness was tested by adhesion assay. Analyses of subcellular distribution of filamentous actin (F-actin) and RhoA in apical and basal cell poles were performed by confocal laser scanning microscopy (CLSM) with and without binding of JAR spheroids as well as with and without inhibition of small Rho GTPases by Clostridium difficile toxin A (toxin A). In the latter case, subcellular distribution of RhoA was additionally investigated by Western blotting.

**Results:**

Ishikawa cells express apical adhesiveness for JAR spheroids and moderate apico-basal polarity. Without contact to JAR spheroids, significantly higher signalling intensities of F-actin and RhoA were found at the basal as compared to the apical poles in Ishikawa cells. RhoA was equally distributed between the membrane fraction and the cytosol fraction. Levels of F-actin and RhoA signals became equalised in the apical and basal regions upon contact to JAR spheroids. After inhibition of Rho GTPases, Ishikawa cells remained adhesive for JAR spheroids, the gradient of fluorescence signals of F-actin and RhoA was maintained while the amount of RhoA was reduced in the cytosolic fraction with a comparable increase in the membrane fraction.

**Conclusion:**

Ishikawa cells respond to JAR contact as well as to treatment with toxin A with rearrangement of F-actin and small GTPase RhoA but seem to be able to modify signalling pathways in a way not elucidated so far in endometrial cells. This ability may be linked to the degree of polar organisation observed in Ishikawa cells indicating an essential role of cell phenotype modification in apical adhesiveness of uterine epithelium for trophoblast in vivo.

## Background

Human embryo implantation is a complex sequence of events which starts with adhesion of the blastocyst to the endometrial lining of the receptive uterus. In order to permit adhesion, uterine epithelial cells develop competence to bind trophoblast to their apical (free) poles [reviewed by [1-4]]. This receptive state of uterine cells is marked by numerous cellular modifications including a reduced thickness of the glycocalyx and a changed composition of proteins and glycoproteins in their apical plasma membrane [5-8]. Also the lateral and basal compartments of these cells are involved in those transformations showing e.g. increased depth and geometrical complexity of tight junctions laterally and a reduced contact to the substratum at the basal cell poles [reviewed by [2]]. Additionally, organisation of the cell architecture and signalling systems seem to be changed [3,9-16]. These modifications point to a reduction or destabilisation of apico-basal polarity of uterine epithelial cells in preparation for trophoblast adhesion during early embryo implantation [2,17-19].

In order to get more detailed insight into molecular processes in uterine epithelial cells when in contact with trophoblast, we have established an in vitro model simulating this cell-to-cell contact [20-22]. We use multicellular spheroids of human trophoblastoid JAR cells as a model for blastocyst trophoblast. These spheroids are delivered onto monolayers of human uterine epithelial RL95-2 cells serving as a model for the receptive uterine epithelium. We previously demonstrated that formation of stable cell-to-cell bonds depends in this model on RL95-2 cells' actin cytoskeleton (F-actin) and small GTPases of the Rho family, most likely RhoA. Data suggested that activation of Rho GTPases and co-ordinated rearrangement of F-actin within the apical and the basal poles of uterine epithelial cells in response to trophoblast binding are part of a generalised structural and functional reorganisation of the cellular architecture of uterine epithelial cells [23-25].

Although RL95-2 cells have proven a useful tool for these investigations, e.g. concerning the interaction between signal transduction events and cytoskeleton [14,23], this model can be criticised since these cells stably and rigidly express a non-polar epithelial phenotype [21,22]. Thus, they do not mimic exactly the in vivo uterine epithelium which destabilises and partially down-regulates (but nevertheless maintains a degree of) apico-basal polarity at receptivity. Therefore, an optimised model should consist of a human uterine epithelial cell line that has maintained polar organisation to a higher degree than RL95-2 cells but permits trophoblast attachment in order to simulate the in vivo situation more closely. As shown in the present communication, Ishikawa cells [26] meet these criteria at least to a certain extent. In addition, this cell line represents a possible alternative model for processes involved in human embryo implantation [27-30].

Data show that Ishikawa cells respond to JAR contact as well as to treatment with toxin A with rearrangement of F-actin and a redistribution of the small GTPase RhoA. Nevertheless, this reorganisation differs in various aspects from the characteristics of reaction known from other endometrial model cells investigated so far, e.g. RL95-2 cells [24,25]. This is probably due to the expression of a specific polar phenotype by Ishikawa cells indicating an essential role of cell phenotype modification in apical adhesiveness of uterine epithelium for trophoblast in vivo.

## Methods

### Antibodies and fluorescent dyes

Mouse monoclonal antibody against RhoA (26CH:sc-418) was purchased from Santa Cruz Biotechnology (Heidelberg, Germany). Mouse monoclonal antibody against E-Cadherin (C20820) was obtained from Transduction Laboratories (Heidelberg, Germany). Mouse monoclonal antibody against α_v _integrin subunit (0770) was obtained from Dianova (Hamburg, Germany). 5-Chloromethylfluorescein diacetate (CMFDA, Cell Tracker Green; C-2925), the secondary antibodies AlexaFluor 488 goat anti-mouse IgG (A-11001), AlexaFluor 488 goat anti-rat IgG (A-11006) and AlexaFluor 633 goat anti-mouse IgG (A-21052) were obtained from Molecular Probes/MoBiTec (Goettingen, Germany). Tetramethylrhodamine isothiocyanate (TRITC)-conjugated phalloidin was obtained from Sigma (Taufkirchen, Germany).

### Cell culture

The human endometrial cancer cell line Ishikawa was derived from an endometrial adenocarcinoma [26] and was cultured as previously described by the group of Schulz [31,32] from which the cells were obtained. The cells were maintained in MEM Dulbecco medium (Gibco, Eggenstein, Germany) supplemented with 15% FCS, 5 ml L-glutamine, 100 U/ml penicillin and 100 μg/ml streptomycin (Boehringer, Mannheim, Germany). For electron microscopical experiments, cells were grown on poly-D-lysine-coated thermanox coverslips. For immunohistochemical stainings poly-D-lysine-coated glass coverslips were used as described previously [20,21]. As an invasive trophoblast model, multicellular spheroids of human choriocarcinoma JAR cells (ATCC: HTB 144) [33] were placed onto the free surface of endometrial cell monolayers. JAR spheroids were prepared as described previously [20], i.e. a suspension of 450,000 JAR cells per 6 ml RPMI 1640 medium (Gibco, Eggenstein, Germany) supplemented with 10% fetal calf serum (FCS) was agitated at 37°C on a gyratory shaker (Certomat R; Braun, Melsungen, Germany) at 110 rpm in order to form multicellular spheroids 72 h after initiation of culture.

### Attachment assay

The JAR cell attachment assay was performed as described previously [20] with the following modifications [24]: In brief, JAR spheroids were stained with the fluorescent vital dye 5-chloromethylfluorescein diacetate (CMFDA) for 45 min at 37°C on a gyratory shaker. After incubation in JAR growth medium without CMFDA, spheroids were gently delivered onto confluent monolayers of endometrial Ishikawa cells. Poly-D-lysine-coated glass coverslips were taken as controls. After 60 min of co-culture in JAR medium at 37°C and 5% CO_2_, spheroid adhesion to the monolayers was quantified by centrifugation of the coverslips at 12 g for 5 min with spheroid surface facing down. As a measure for adhesiveness of monolayers, attached spheroids were counted and expressed as a percentage of the number of spheroids seeded initially.

To inactivate small GTPases of the Rho family, confluent monolayers of Ishikawa cells were incubated with Clostridium difficile toxin A [34] for 24 h at a concentration of 100 ng/ml in growth medium. Then, the attachment assay was performed as described above. Controls were cultured for 24 h without toxin A.

### Preparation of subcellular fractions and Western blotting

Ishikawa cells were suspended in ice-cold buffer A (20 mM Tris/HCl, pH 7.4, 2 mM EDTA, 1 mM EGTA, 1 mM dithiothreitol, 1 mM phenylmethylsulphonylfluoride, 50 μg/ml soybean trypsin inhibitor, 10 μM pepstatin, 10 μM leupeptin and 2 μg/ml aprotinin) and thereafter disrupted by three cycles of freeze-thawing, using liquid nitrogen and a 37°C water bath. The resulting lysates were centrifuged at 17,000 × g for 5 min, thereby separated into the cytosolic fraction and the pellet. The latter was resuspended in ice-cold buffer A, supplemented with 1 % Triton X-100, sonicated 5 times for 10 s each and centrifuged as above, to produce in the cytoskeletal fraction [24]. The samples were boiled for 5 min in Laemmli buffer and proteins were separated by SDS-PAGE on 12.5 % acrylamide gels (per lane: lysate 10 μg, membrane and cytosolic fraction as well as cytosol 100 μg each), transferred to nitrocellulose membranes, and stained with an specific anti-RhoA antibody (dilution 1:1000; 1 h incubation). The membranes were then incubated with secondary antibody, and immunoreactivity was visualised by enhanced chemiluminescence (Amersham Pharmacia Biotech, Freiburg, Germany) as described before [24].

### Immunofluorescence and F-actin staining

Immunostaining was performed as described before [24,25]. Samples were fixed and permeabilised by a 1 + 1 mixture of ethanol-acetone for 10 min at room temperature. After rinsing, non-specific binding sites were blocked by incubation with 0.5% bovine serum albumine (BSA) in phosphate buffered saline (PBS) for 15 min. Then, samples were incubated for 90 min at 37°C with the primary antibody (see above), which was omitted in control stainings. Thereafter, cells were rinsed in PBS/0.5% BSA, incubated with the corresponding fluorescence-conjugated secondary antibody (see above) for 90 min at 37°C, again rinsed and mounted in PBS supplemented with 90% glycerol and 1% p-phenylendiamine. For staining of F-actin, samples were fixed with 3% paraformaldehyde for 15 min at room temperature, permeabilised by incubation with 0.05% Triton X-100 for 2 min and then incubated for 15 min with TRITC-phalloidin which was omitted in controls. Afterwards, samples were mounted in PBS/glycerol/phenylendiamine.

In order to combine immunofluorescence and F-actin staining, samples were fixed with 3% paraformaldehyde for 15 min at room temperature and permeabilised in Triton X-100 according to the F-actin staining protocol. Then, primary antibody and TRITC-phalloidin were applied simultaneously for 90 min at 37°C. Both were omitted in controls. Thereafter, the immunostaining reaction was performed as described above. All samples were examined by confocal laser scanning microscopy.

### Confocal laser scanning microscopy, image analysis and processing

Confocal microscopy was performed using a Zeiss Axiovert 100 M microscope attached to a confocal laser scanning microscopy system (CLSM) (model LSM 510; Carl Zeiss, Jena, Germany) as described previously [24]. As excitation sources, an argon laser with output at 488 nm and two helium-neon lasers with output at 543 and 633 nm, respectively, were available. Fluorescence emission of CMFDA was encoded as 'blue' after passing a 505–530 nm bandpass filter, emission of TRITC as 'red' after passing a 560–565 nm bandpass filter, and emission of Alexa Fluor 633 and Alexa Fluor 488 as 'green' after passing a 650 nm longpass filter and a 505–530 nm bandpass filter, respectively. Optical tomography was performed at 0.5 μm intervals using a 40-fold oil immersion objective with a numerical aperture of 1.3 NA and a pinhole size corresponding to a value of 1.0 of the airy disk. To improve the signal to noise ratio, each slice was scanned 8 times followed by averaging.

All measurements were performed on several single Ishikawa cells. In monolayers without contact to JAR cells, examined Ishikawa cells were selected randomly from the entire photographic field. In monolayers with contact to JAR spheroids, only Ishikawa cells with definite membrane contact to JAR cells were included. Mismatching with JAR cells was avoided by pre-labelling those with CMFDA. To obtain semi-quantitative data, intensities of fluorescence signals of each channel were measured and expressed as average grey scale values (gsv) using the CLSM software (version 2.8 SR 1; Carl Zeiss). The apical-most and the basal-most slices of each stack were selected for analysis. Image Pro Plus software was used for image enhancement as well as spatial measurements of RhoA-positive granules (version 4.5; Media Cybernatics, Crofton, Md., USA). It was additionally equipped with a Gaussian filter module [35] and a homomorphic filter plugin [36]. Adobe Photoshop software (version 7.0; Adobe Systems, San Jose, Calif., USA) was used for the arrangement of RGB-colour images out of single grey scale images each representing the signal of one colour channel.

### Transmission electron microscopy

Cells were grown as monolayers on thermanox coverslips as described above, rinsed twice in PBS and fixed in 2.5% glutaraldehyde in 0.1 M cacodylate buffer, pH 7.4, for 30 min at room temperature. After several washings in cacodylate buffer, samples were postfixed with 1% OsO_4 _in cacodylate buffer, dehydrated with graded ethanol and propylene oxide, and embedded in epoxy resin mixture [37]. The embedded monolayers were separated from the thermanox coverslip by short-term heating on a hot plate. Ultrathin sections were mounted on 200-mesh copper grids, double-stained with uranyl acetate and lead citrate and examined with a Zeiss 902 A at 80 kV (Carl Zeiss, Jena, Germany).

### Statistical analysis

In Fig. 4, data are presented as medians (first – third quartile) with *n *denoting the number of experiments. Statistical analysis was performed by using both the Kruskal-Wallis test for global differences between groups and the Wilcoxon Signed Rank Sum test for their pairwise comparison. A value of p < 0.05 was considered significant. In Fig. 6, data are presented as means ± SEM with *n *denoting the number of experiments. Paired and unpaired t-tests were applied as appropriate. A value of p < 0.05 was considered significant.

## Results

### I. Morphology of Ishikawa monolayers

#### Ultrastructural features and localisation of adhesion molecules

**Figure 1:**
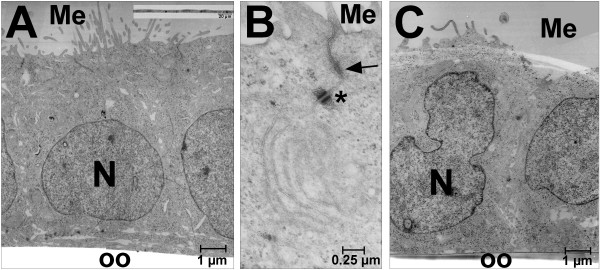
**Ultrathin sections of Ishikawa cells. A**: Ishikawa cells before treatment with toxin A. Cells grow as monolayers and show apico-basal polarity with nuclei located at the base of the cells and organelles found predominantly in the supranuclear region of the cells. Insert (light microscopy, cross section) shows overview of Ishikawa cells growing as monolayers. **B**: Lateral cell membranes show tight junctions, adherens junctions and desmosomes in varying combinations, whereas regular junctional complexes consisting of tight junction, adherens junction and desmosomes in apico-basal sequence were rarely seen. **C**: Ishikawa monolayers after treatment with toxin A. Standard electron microscopy showed no substantial differences between untreated (A) and toxin A-treated cells (C). Me: cell culture medium; N: nucleus; oo: coverslip; *: desmosome; arrow: adherens junction.

**Figure 2:**
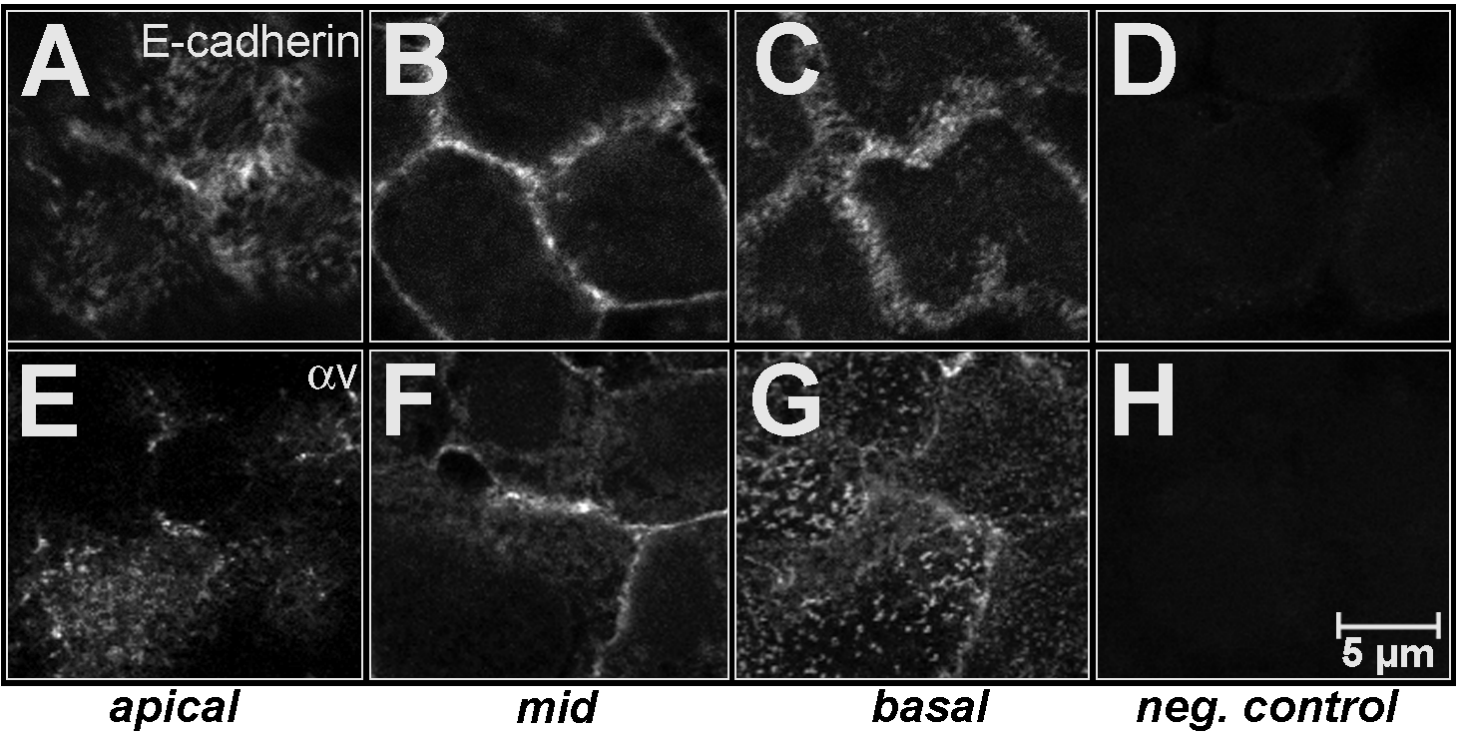
**Localisation of E-cadherin and integrin subunit α_v_. A-D**: E-cadherin; **E-H**: integrin subunit α_v_, both detected by confocal laser scanning microscopy. Note that both adhesion molecules are located in all plasma membrane domains, including the apical one. Typical patterns are presented for apical (apical) and basal (basal) cell poles as well as for the middle part (mid) of cells. D,H: negative controls.

Ishikawa cells typically grow as monolayers as demonstrated by electron microscopy (Fig. 1). Individual cells showed a uniform size and a cylindrical cell shape. Nuclei were located predominantly in the basal compartment of the cells. Endoplasmatic reticulum, Golgi apparatus and mitochondria were arranged predominantly in the supranuclear region of the cells (Fig. 1A). At their apical poles, membrane protrusions were found which varied in length and shape. Laterally (Fig. 1B), tight junctions were located in the subapical regions and numerous interdigitations of adjacent membranes were seen. Additionally, adherens junctions and desmosomes were scattered along the lateral membranes whereas regular junctional complexes consisting of tight junction, adherens junction and desmosomes in apico-basal sequence were rarely found. As shown by immunohistochemistry, E-cadherin (Fig. 2A–D) and integrin subunits α_v _(Fig. 2E–H) as well as β_1 _(data not shown) were detected in all regions of the plasma membrane of Ishikawa cells, including their apical membrane (Fig. 2A,E).

#### F-actin and RhoA

**Figure 3:**
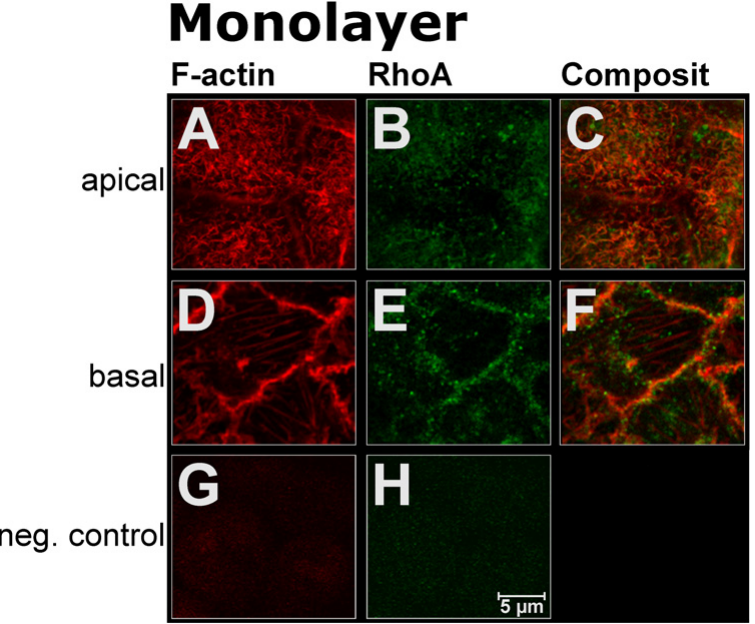
**Localisation of F-actin and RhoA in Ishikawa monolayers before binding of JAR spheroids. A, D**: F-actin (red); **B, E**: RhoA (green); **C, F**: Merger of F-actin and RhoA signal. xy-sections representing second slices from the apical (apical) and basal (basal) cell poles, respectively. Typical patterns are presented. **G, H**: negative controls.

**Figure 4:**
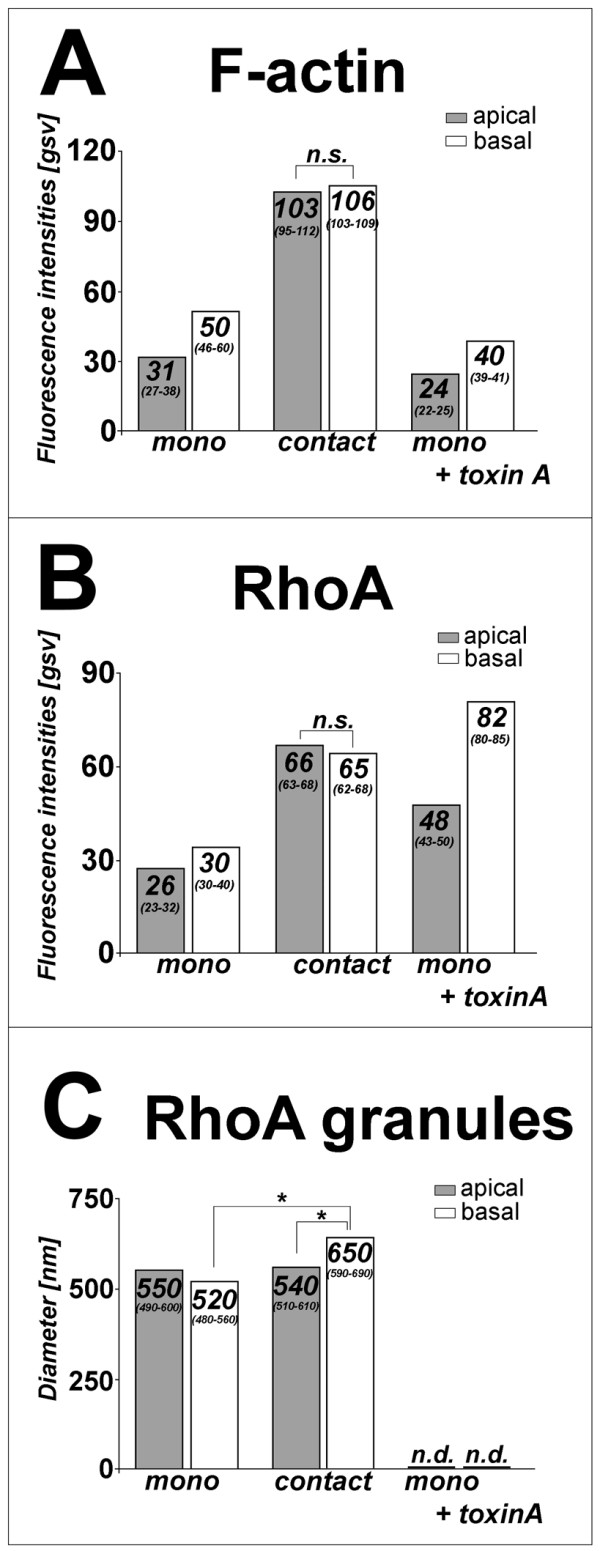
**Quantification of F-actin and RhoA. A: **Quantification of F-actin in the apical and basal regions of monolayer cultured Ishikawa cells before (mono) and after (contact) binding of JAR spheroids as well as after treatment with toxin A (mono + toxin A). For semi-quantitative evaluation of fluorescence, grey scale values (gsv) of each colour channel were determined within double-labelled cells. Stacks of 6 *xy*-sections at 0.5 μm intervals were collected with the first marked slice at the apical cell surface and basal cell pole, respectively. Numbers of Ishikawa cells tested: *n *= 14 (mono), *n *= 12 (contact), *n *= 15 (mono + toxin A). Values differ significantly (p < 0.05) between these experimental groups but not between apical and basal cell poles within Ishikawa cells being in contact with a JAR spheroid (n.s.). Data are presented as medians (first – third quartile). **B: **Quantification of RhoA in the apical and basal regions of monolayer cultured Ishikawa cells before (mono) and after (contact) binding of JAR spheroids as well as after treatment with toxin A (mono + toxin A). Semi-quantitative evaluation of fluorescence was done as described under A. Values differ significantly (p < 0.05) between these experimental groups but not between apical and basal cell poles within Ishikawa cells being in contact with a JAR spheroid (n.s.). Data are presented as medians (first – third quartile). **C: **Diameter of RhoA-positive granules in Ishikawa cells before (mono) and after contact (contact) with JAR spheroids, number of cells tested: *n *= 14 (mono), *n *= 12 (contact). Values differ significantly (p < 0.05) between apical and basal cell poles in contact situation as well as between basal cell poles in contact vs. non-contact situation (*). n.d.: not detectable. Data are presented as medians (first – third quartile).

Staining of F-actin revealed a small band of strong fluorescence signal in the periphery of the cells (Fig. 3A,D). Small F-actin-positive aggregates were observed apically corresponding in size and shape to membrane protrusions seen in electron microscopy (see above). Numerous stress fibres and focal contacts were detected in the basal cell poles (Fig. 3D). In addition, the cytoplasm stained weakly. The intensity of F-actin staining was 1.6x higher in the basal compared to the apical cell poles of Ishikawa cells (31 (27–38) gsv apically vs. 50 (46–60) gsv basally; Fig. 4A).

**Figure 5:**
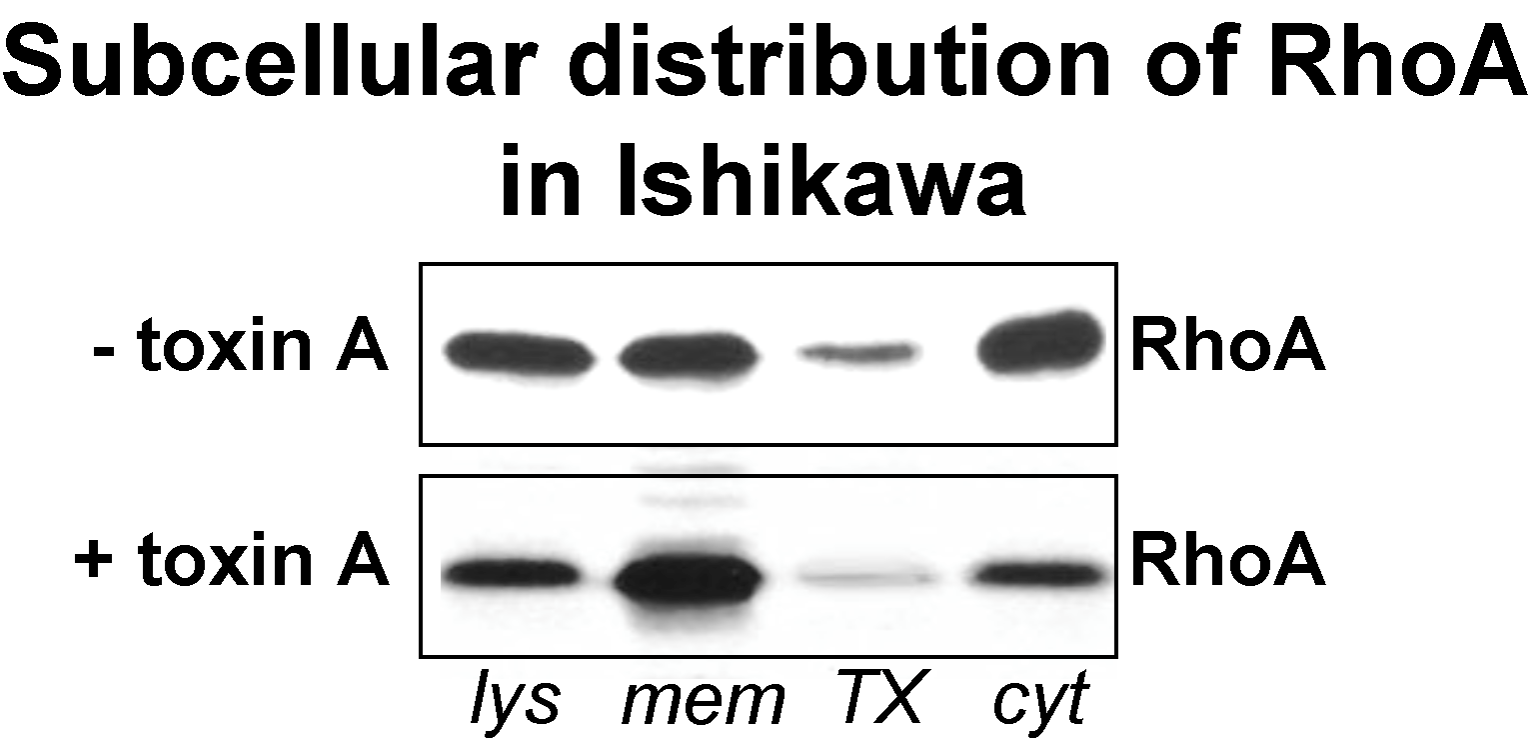
**Immunoblot analysis of endogenously expressed RhoA. **Ishikawa monolayers remained untreated (- toxin A) or were treated with 100 ng/ml toxin A for 24 h (+ toxin A). Preparation of cell lysates (lys), membranes (mem), cytoskeleton fraction (TX) and cytosol (cyt) of cells was performed as described. Proteins (lysates: 10 μg/lane; membranes, cytoskeleton fraction, cytosol: each 100 μg/lane) were separated by SDS-PAGE and subsequently immunoblotted. Data shown are typical for three independent experiments.

In Ishikawa cells, measurement of RhoA fluorescence intensity revealed a 1.2x higher signal in the basal compared to the apical cell poles (26 (23–32) gsv apically vs. 30 (30–40) gsv basally; Fig. 4B). A granular staining pattern was seen with single bigger granules within dominating fine-grained signals (Fig. 3B,E). These granules showed a diameter of apically 550 (490–600) nm and of basally 520 (480–560) nm (Fig. 4C) and were arranged preferentially in the periphery of the cells and along the F-actin aggregates (Fig. 3C,F). Western blotting showed a relatively homogeneous distribution between membrane fraction and cytosol whereas the cytoskeletal fraction was nearly free of RhoA (Fig. 5).

**Figure 6:**
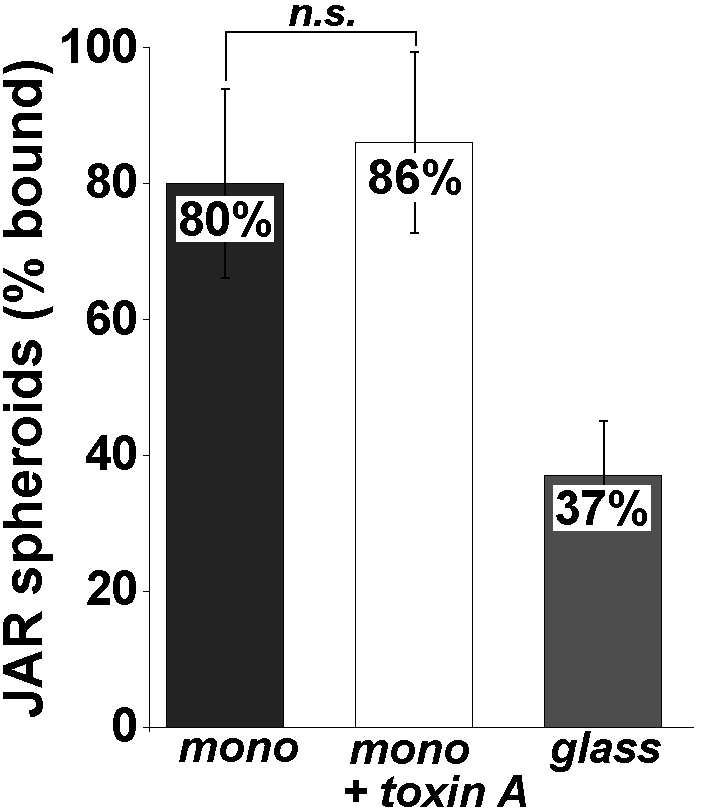
**Apical adhesiveness of Ishikawa cells. **Apical adhesiveness for JAR cell spheroids of monolayer-cultured Ishikawa cells was determined in the centrifugal force-based adhesion assay before (mono; *n *= 349) and after (mono + toxin A; *n *= 358) treatment with Clostridium difficile toxin A. For comparison, adhesiveness of poly-D-lysine-coated glass coverslips (glass) for spheroids is shown (*n *= 335). Values differ significantly (p < 0.05) between experimental groups except for Ishikawa cells treated with and without toxin A (n.s.). Data are presented as means ± SEM.

### II. Molecular mechanisms of apical adhesiveness of Ishikawa monolayers for JAR spheroids

#### Adhesiveness for JAR spheroids

In order to determine adhesiveness of the apical cell poles of Ishikawa cells for trophoblastoid cells, multicellular spheroids of JAR cells were placed onto confluent monolayers of Ishikawa cells. As shown in Fig. 6, the majority of seeded JAR spheroids adhered to the Ishikawa monolayers (80 ± 14 %). 37 ± 8 % of JAR spheroids adhered to poly-D-lysin-coated glass coverslips serving as controls. Therefore, significantly more JAR spheroids adhered to Ishikawa monolayers as compared to controls, i.e. Ishikawa cell monolayers permitted JAR adhesion.

#### Morphology of the contact site

**Figure 7:**
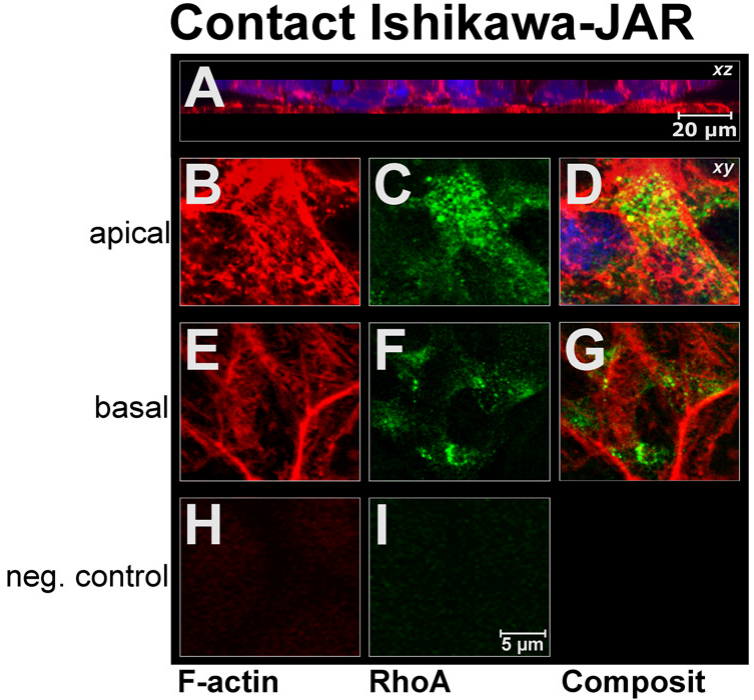
**Localisation of F-actin and RhoA in Ishikawa monolayers after binding of JAR spheroids. A**: xz-section through contact site of Ishikawa cells and JAR spheroid after tracking of JAR cells with the vital dye CMFDA (blue). red: F-actin cytoskeleton. **B**-I: Localisation of F-actin (red) (B, E) and RhoA (green) (C, F) in the apical (apical) and basal (basal) regions of Ishikawa monolayers after binding of JAR spheroids tracked with CMFDA (blue). xy-sections represent second slice from the apical and basal cell poles, respectively. D, G: Merger of F-actin and RhoA signal. Typical patterns are presented. H, I: negative controls.

Morphology and cytoskeletal details of the contact site was examined by fluorescence confocal microscopy after tracking JAR spheroids with CMFDA and staining of F-actin in Ishikawa cells and JAR cells with phalloidin-TRITC (Fig. 7A). CLSM revealed that a large membrane contact area was formed between the JAR spheroid and the Ishikawa monolayer. All cells of the lower part of the spheroid that were exposed to the monolayer and all adjacent cells of the latter participated in the adhesion interaction.

#### F-actin and RhoA at the contact site

After confrontation culture of Ishikawa monolayers and JAR spheroids (Fig. 7A) the distribution of F-actin and of RhoA was examined by CLSM in detail (Fig. 7B–I). The asymmetric distribution of F-actin along the apico-basal axis found in Ishikawa monolayers without contact to JAR spheroids disappeared in the contact area. The distinct F-actin aggregates observed in the apical poles of non-contact Ishikawa cells were not detectable any longer but an increased amount of stress fibres basally as well as of homogeneous staining was seen (Fig. 7B,E). Thus, a significant increase in fluorescence intensity was detected in the contact zone with regard to F-actin, i.e. 103 (95–112) gsv apically and 106 (103–109) gsv basally when compared to values before contact (Fig. 4A).

The apico-basal asymmetry seen in the pre-contact situation was lost after contact also with respect to RhoA (Fig. 7C,F). The overall signal of RhoA increased significantly in the apical and basal regions of the cells after attachment of JAR spheroids, i.e. 66 (63–68) gsv apically vs. 65 (62–68) gsv basally (Fig. 4B). Also the diameter of RhoA-positive granules increased significantly at the basal cell poles from 520 (480–560) nm to 650 (590–690) nm in the contact area (Fig. 4C). Apically, the mean diameter (540 (510–610) nm) did not differ significantly from the non-contact situation (550 (490–600) nm; Fig. 4C).

#### Ishikawa monolayers after treatment with Clostridium difficile toxin A

In order to determine the role of RhoA for adhesiveness of Ishikawa cells, Rho GTPases were inhibited by treatment with Clostridium difficile toxin A (toxin A). Standard electron microscopy showed no substantial difference between untreated (Fig. 1A) and toxin A-treated cells (Fig. 1C).

Upon treatment, F-actin fluorescence decreased significantly to 24 (22–25) gsv in the apical and 40 (39–41) gsv in the basal cell poles. (Fig. 4A). In contrast, RhoA intensities increased significantly in the apical as well as in the basal cell poles (after treatment: 48 (43–50) gsv apically vs. 82 (80–85) gsv basally; Fig. 4B). A changed subcellular distribution of RhoA was found by Western blotting (Fig. 5). The amount of RhoA was reduced in cytosolic fraction with a comparable increase in the membrane fraction while the cytoskeleton fraction remained free of detectable RhoA. After incubation of Ishikawa cells for 24 h with 100 ng/ml toxin A and subsequent attachment assay, 86 ± 15 % of seeded JAR spheroids adhered to the monolayers (Fig. 6). This was no significant difference to untreated monolayers where 80 ± 14 % of JAR spheroids adhered.

## Discussion

The formation of stable cell-to-cell bonds to trophoblast cells requires a rearrangement of the cytoskeleton (F-actin) and redistribution of small GTPases of the Rho family in endometrial cells [23-25]. Consistent with this, F-actin and RhoA increase significantly in Ishikawa cells being in contact with JAR spheroids. This may be part of a generalised structural and functional reorganisation of the cellular architecture as the co-ordinated rearrangement is observed in the apical as well as in the basal cell poles.

Interestingly, Ishikawa cells equalise F-actin and RhoA signals in the apical and basal cell poles in response to JAR contact. This is in contrast to another endometrial cell line, e.g. RL95-2, which inverts the gradient between apical and basal cell poles as shown previously [25]. This identical trend but different extent of modification of apico-basal polarity might be due to differences between Ishikawa and RL95-2 cells in the initial subcellular distribution of RhoA found before JAR contact: in Ishikawa cells, large amounts of RhoA were detected in the membrane fraction as well as in the cytosol but in RL95-2 considerable quantities of RhoA were only seen in the membrane fraction [24]. These differences may even indicate that distinct signalling pathways are involved which comprise a specific subset of RhoA-specific guanine nucleotide exchange factors (GEFs), trigger GTP loading, and thus activation of RhoA [38,39]. Indeed, RhoA and its GEFs have been reported to be present in caveolae and lipid rafts. These compartmentalised membrane signalling domains are believed to confer specificity to the complex mechanisms of RhoA signalling [39,40]. The fact that Ishikawa cells are not quite as adhesive for JAR spheroids as RL95-2 cells may be based on such differences [21].

After treatment with toxin A which inhibits the family of small Rho GTPases, F-actin decreases significantly in apical and basal poles of Ishikawa cells while RhoA increases significantly. In addition, adhesiveness of Ishikawa cells to JAR spheroids is not affected. Regarding this, one could argue that toxin A uptake is insufficient in Ishikawa cells but marked changes in F-actin staining with significant decrease in fluorescence intensity as well as changes in distribution of RhoA clearly contradict this. A hypothetical possibility might be that Ishikawa cells use alternative signalling cascades which compensate inhibition of small Rho GTPases. This may include an increased synthesis of Rho proteins at a level which cannot prevent morphological changes but may allow to maintain special signalling cascades resulting in preserved adhesiveness for JAR spheroids. Additionally, Ishikawa cells may be able to replace small GTPases of the Rho family by other members of the superfamily of GTPases. Indeed, it is established that different Rho GTPases can influence each other's activities [38-41]. It has been even proposed recently that Rho signalling may have profound effects on Rap signalling, the latter known to modulate the organisation of the actin cytoskeleton as well [42-44].

The response of Ishikawa cells to toxin A is in contrast to the previously described behaviour of RL-95-2 cells which show a different distribution of RhoA in Western blotting after treatment with toxin A as compared to Ishikawa cells [24]. Furthermore, adhesiveness for JAR spheroids is lost in RL95-2 cells after toxin A treatment. These differences may be due to alternative signalling cascades which may also be responsible for the different degree of apico-basal polarity in both cell lines. Indeed, Ishikawa cells have preserved major aspects of apico-basal polarity typical for simple epithelia as shown here. Nevertheless, the modified composition of junctional complexes as well as the generalised membrane localisation of E-cadherin and integrins imply a slight downregulation of the polar epithelial phenotype [19]. These modifications may be a prerequisite for the fact that also Ishikawa cells do react to JAR cell binding with molecular reorganisation in the apical as well as in the basal regions. In further investigations, it would be interesting to use a model system that mimics the in vivo situation even closer. Immortalised endometrial cells may be an appropriate experimental model in this respect [e.g. [45]].

Our findings indicate that the host cell response is indeed a complex process involving a reorganisation of the whole cell architecture [17,18] and not just the apical cell pole as suggested by concepts focussing on the apical plasma membrane [12]. It remains to be seen whether and to what extent this process may be governed by the same type of regulating genes and cellular mechanisms as involved in epithelial fusion processes during development and epithelial-mesenchymal transitions (EMT) [2,17]. In particular, the postulated master genes and specific signalling cascades studied in connection with complete EMT processes [46-50] should be of interest here.

## Conclusion

Data presented here support the hypothesis that the actin cytoskeleton and the small GTPase RhoA play an important role in embryo implantation. As toxin A cannot prevent attachment of JAR spheroids to Ishikawa cells, these cells seem to be able to modify signalling pathways in a way not elucidated so far in endometrial cells. This ability may be linked to the peculiar polar epithelial phenotype observed in Ishikawa cells. This is consistent with the notion that the extent of polar organisation of uterine epithelial cell lines influences their responses to JAR cell contact implying an essential role of cell phenotype modification in apical adhesiveness of uterine epithelium for trophoblast.

## Competing interests

The author(s) declare that they have no competing interests.

## Authors' contributions

CH conceived of the study and carried out the confocal laser scanning microscopical and electron microscopical investigations. MS provided expertise in small Rho GTPases and conducted biochemical experiments. HWD participated in the design of the study and helped to draft the manuscript. MT conceived of the study, and participated in its design and coordination and helped to draft the manuscript. All authors read and approved the final manuscript.
